# Analysis of 108 flexible bronchoscopies for the removal of foreign bodies from the airways

**DOI:** 10.31744/einstein_journal/2023AO0391

**Published:** 2023-12-01

**Authors:** Altair da Silva Costa, Addy Lidvina Mejia Palomino, Iunis Suzuki, Rodrigo Gobbo Garcia, Paulo Rogerio Scordamaglio, Marcelo Gervilla Gregorio, Felipe Nominando Diniz Oliveira, Manoel Ernesto Peçanha Gonçalves, Marcia Jacomelli

**Affiliations:** 1 Hospital Israelita Albert Einstein São Paulo SP Brazil Hospital Israelita Albert Einstein , São Paulo , SP , Brazil .; 2 A.C.Camargo Cancer Center São Paulo SP Brazil A.C.Camargo Cancer Center , São Paulo , SP , Brazil .

**Keywords:** Bronchoscopy, Respiratory aspiration, Asfixia, Airway obstruction, Children, Adults, Therapeutics, Diagnosis

## Abstract

**Objective:**

To describe the clinical, bronchoscopic, diagnostic, and therapeutic aspects between children and adults.

**Methods:**

This retrospective study compared the clinical and bronchoscopic characteristics of adults and children who underwent bronchoscopy for suspected foreign body aspiration. Data on sex, outpatient or emergency origin, bronchoscopy results, characteristics of the aspirated foreign body, and complications were analyzed.

**Results:**

In total, 108 patients were included in the analysis, with foreign body aspiration diagnosed in 69% of patients (30 children and 44 adults). In 91% of patients, there was a clinical history suggestive of aspiration. The mean age of the adults was 65.89 (±19.75) years, and that of the children was 2.28 (±1.78) years. Most of the children were under 3 years of age (80%), while adults were mostly 70 years of age or older (54.5%). Emergency care was more common among children than adults. The most common foreign bodies found in both age groups were organic bodies, primarily seeds. The most frequent locations of foreign bodies were the lobar bronchi in adults and the main bronchi in children. Flexible bronchoscopy is the primary method for diagnosis and treatment. Transient hypoxemia occurred particularly frequently in children (5%).

**Conclusion:**

Foreign body aspiration, particularly that involving seeds, is more common in the extremes of age. A clinical history suggestive of aspiration is crucial in determining the need for bronchoscopy, which should be performed as early as possible. Flexible bronchoscopy is an effective and safe diagnostic technique.

## Summary

In Brief

Foreign body aspiration is common in pediatric and adult patients and can result in coughing, wheezing, dyspnea, and other respiratory symptoms. The clinical history should be valued more than imaging exams such as radiography and tomography.

Bronchoscopy under anesthesia is the gold standard for diagnosis and treatment, providing good visualization, secure removal of the foreign body with timely relief and prevention of complications.

## Abstract (key points)

Highlights

Foreign body aspiration into the airways is common in adults and children, especially in the extremes of age.

Coughing, wheezing, and dyspnea may occur and can be life-threatening.۪In airway foreign body, clinical suspicion is more important than imaging exams.

Bronchoscopy under general anesthesia is safe and effective for removing foreign bodies; it ensures timely relief and prevents further complications

## Graphical abstract

**Figure:**
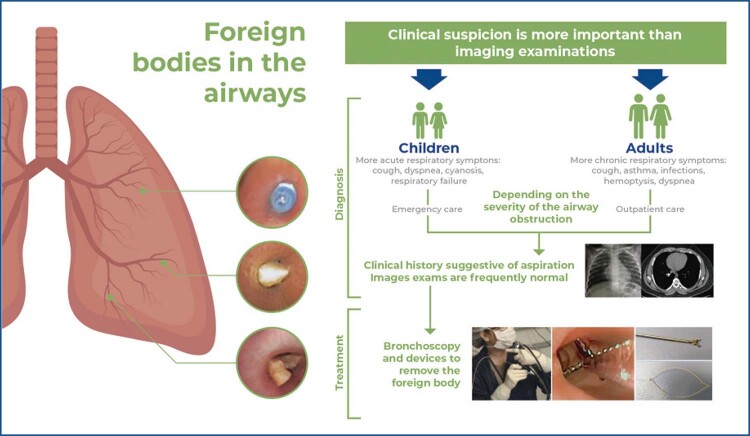

## INTRODUCTION

In 1897, Dr. Gustav Killian performed the first bronchoscopy to remove a bone fragment from the left main bronchus. The procedure utilized an esophagoscope and a frontal illumination source. In 1905, Dr. Chevalier Jackson improved the rigid bronchoscope by incorporating a light metal tube with a connector for ventilation and suction channels, which is still used today. ^( 1 )^ The invention of flexible bronchoscopy by Dr. Shigeto Ikeda in 1966 marked a significant milestone in advancing diagnostic and therapeutic techniques for various respiratory diseases. ^( 1 , 2 )^

Foreign body (FB) aspiration into the airways is a critical event that poses a risk of immediate death, particularly in children, and can lead to chronic complications, such as recurrent infections, hemorrhages, and chronic cough. The prevalence of FB aspiration was 8.3 individuals per 100,000 inhabitants, with a mortality rate of 0.6/100,000 inhabitants. ^( 3 )^ Asphyxia is the third leading cause of death among young individuals in Brazil. The estimated cost of treating FB aspiration in the United States is approximately US$13 million. ^( 3 )^

The clinical presentation of aspiration typically includes symptoms such as coughing, choking, dyspnea, and cyanosis. Many people experience mild choking incidents at home, which usually resolve spontaneously without significant clinical consequences. ^( 4 - 6 )^ However, young children are at a higher risk of suffocation due to mechanical obstruction of the airway passage.

Generally, aspiration progresses through three phases. ^( 1 , 7 , 8 )^ The acute phase is characterized by choking, coughing, and cyanosis, with an immediate risk of suffocation and potential death. Subsequently, the accommodation phase ensues, during which the FB settles in a more distal airway, leading to reduced symptoms but persistent coughing, snoring, wheezing, and bronchospasm. The chronic phase involves complications resulting from the persistence of the aspirated material in the airway lumen, which can vary in severity and manifest as bronchospasms, dyspnea, recurrent infections, and hemoptysis. In most cases, diagnosis is made during the first two phases, and clinical suspicion based on historical data plays a crucial role. ^( 1 - 3 , 7 , 8 )^ However, diagnosis may be delayed in some cases, especially in adults.

## OBJECTIVE

To describe the clinical, diagnostic, and therapeutic aspects of bronchoscopy for foreign bodies in the airways of children and adults who underwent bronchoscopy for suspected foreign body aspiration.

## METHODS

This retrospective descriptive study utilized a database from the Bronchoscopy Center of *Hospital Israelita Albert Einstein* in São Paulo, Brazil. The study period spanned 8 years, from January 2014 to August 2022.

Patients who underwent bronchoscopy for suspected FB aspiration were included in the analysis based on their clinical history ( *e.g* ., choking episodes and trailing pneumonia) and chest imaging (radiography or tomography). Patients with incomplete data in the hospital’s electronic medical records system were excluded from the analysis.

The analysis included demographic information such as age, gender, the elective or urgent origin of the bronchoscopy procedure, bronchoscopy findings, characteristics of the aspirated FB in both adults and children, and reported complications.

In this study, only a descriptive analysis was performed, and no inferential calculations were conducted. This study was approved by the *Hospital Israelita Albert Einstein* Ethics Committee through the Brazil CAAE: 52243515.4.0000.0071; # 1.450.042.

## RESULTS

During the analysis period, 115 flexible bronchoscopies were performed by the bronchoscopy team to investigate suspected airway FBs. All procedures adhered to the institutional protocol, which involved general anesthesia administered by an anesthesiologist, the utilization of spontaneous ventilation, and either tracheal intubation or a laryngeal mask.

Of the 115 bronchoscopies conducted, seven were excluded from the analysis because of incomplete system data or the presence of other endoscopic findings, such as secretion plugs or clots. Thus, the final analysis included 108 patients, among whom FBs were confirmed in 69% (n=74) of the patients. Of these, 59% were adults, and 41% were children. In 91% of the cases, the clinical history indicated FB aspiration. In 9% of cases, the presence of an FB was incidentally discovered during bronchoscopy performed for other indications, with a higher occurrence in adults (12%) than in children (3%).

The mean age of adult patients was 65.89 (±19.75) years, while that of children was 2.28 (±1.78) years. Most of the children (n=24; 80%) were under 3 years of age, whereas adults (n=24; 54.5%) were predominantly aged 70 years or older. There was a male predominance in both age groups ( Table 1 ).

**Table 1:** Demographic data and bronchoscopy results in adults and children

Characteristics	Children (n=30)	Adults (n=44)
Age (mean/SD)	2.28 (±1.78)	65.89 (±19.75)
Gender, n (%)		
Male	19 (63.3)	29 (65.9)
Female	11 (36.7)	15 (34.1)
Origin, n (%)		
Emergency unit	14 (46.7)	16 (36.4)
Outpatient (elective)	16 (53.3)	28 (63.6)
Foreign body type, n (%)		
Organic	24 (80)	33 (75)
Inorganic	4 (13.3)	10 (23)
Indefinite	2 (6.7)	1 (2.3)
Foreign body location, n (%)		
Upper airways	6 (20)	9 (20)
Trachea	0	(2.3)
- Right bronchial tree	15 (50)	29 (66)
Main bronchus	10	3
Upper lobe bronchus	1	4
Intermediate bronchus	4	4
Middle lobe bronchus	0	3
Lower lobe Bronchus	0	15
- Left bronchial tree	9 (30)	5 (11.4)
Main bronchus	7	2
Upper lobe Bronchus	0	1
Lower lobe Bronchus	2	1
Complication, n (%)	5 (16.7)	1 (1.35)

For patients with suspected aspiration, whether referred by the emergency department or outpatient clinic, bronchoscopy was performed as soon as possible, considering the necessary fasting time, particularly in children.

Flexible video bronchoscopes of various calibers (ranging from 2.8mm to 6.8mm) and working channels (between 1.2mm and 3.2mm) were utilized. The choice of flexible accessories depends on the bronchoscope channel size and the type of FB that requires grasping. Commonly used flexible accessories include basket-type baskets, serrated flexible forceps, rat-tooth forceps, pelican forceps, and polypectomy handles.

In only one case, a combination of rigid and flexible bronchoscopy was used to facilitate removal and ensure adequate ventilation in a 1-year-old child.

**Figure 1:**
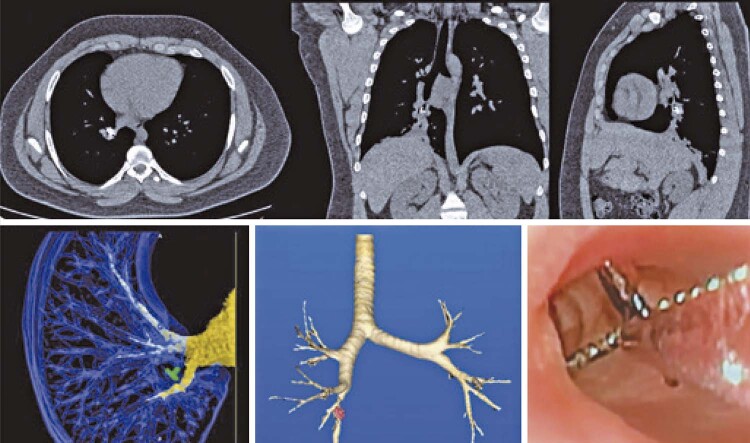
Metallic foreign body in the right lower lobe bronchus 10 years ago – 3D reconstructions and two stage removal

In one adult patient who had a metallic FB in the right lower lobe for 8 years, the following interventions were performed before bronchoscopic removal of the FB: dilatation of scarring stenosis of the lower lobar bronchus using a hydrostatic balloon, electrocauterization, antibiotic treatment, and systemic corticosteroids ( Figure 1 ). The entire process, from the initial bronchoscopy to the FB removal, lasted 15 days.

Figure 1 depicts a metallic FB that remained in the right lower lobe bronchus for 10 years. In the 3D reconstructions, the presence of scarring stenosis was observed. Bronchial dilation and infection treatment were performed, followed by removal (a procedure in two stages).

Predominant involvement of the right bronchial tree was observed in both groups, with the lobe bronchi being more commonly affected in adults (n=25; 56.8%) and the main bronchi in children (n=17; 56.7%).

**Figure 2:**
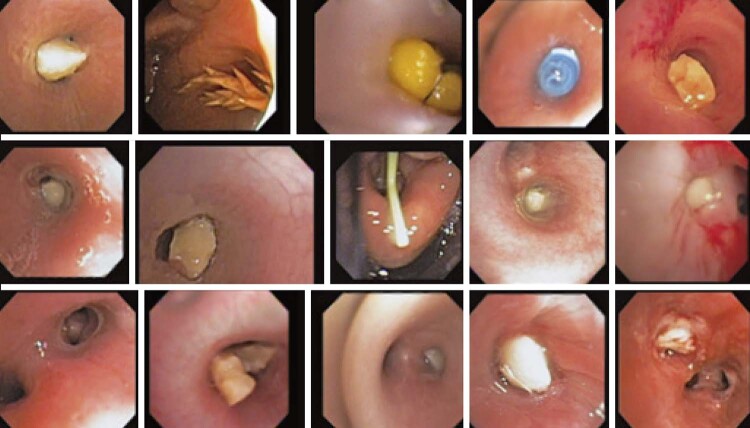
Various foreign bodies – peanuts, twigs, rubber, almonds and corn and others

**Figure 3:**
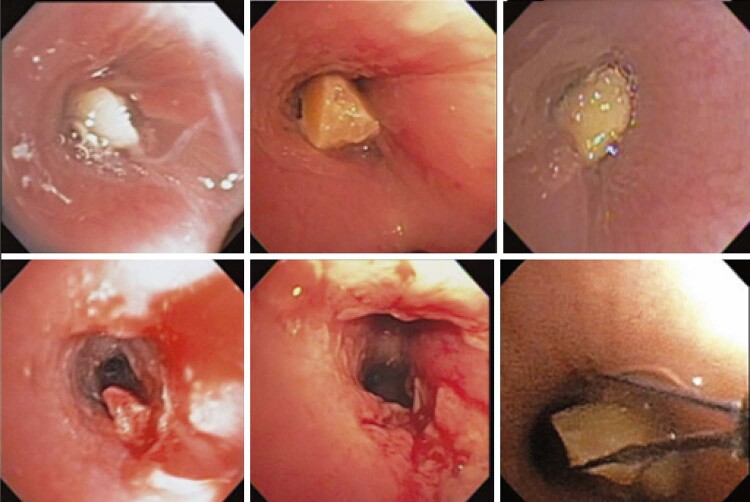
First row - foreign bodies (peanuts), removed with a retractable basket. Second line the immediate appearance after desobstruction

Organic FBs were most frequently encountered in adults and children (Figures 2 and 3). Among these organic FBs, seeds, nuts, and similar items accounted for 70% of the cases in children and 39% in adults ( Table 2 ). Peanuts were the most frequently found FBs in children (n=11; 52%). Conversely, various types of food, such as meat, vegetable fibers, and fish spines, are commonly consumed by adults.

**Table 2:** Description of the foreign bodies aspirated in the two groups

Foreign body types	Adults (n/%)	Children (n/%)
Seed, chestnuts, and similar items	17 (39)	21 (70)
Other foods	13 (29.5)*	1 (3.3) ^#^
Plastic	5 (11.4)	5 (16.7)
Tooth	3 (6.8)	-
Metallic	4 (9)	-
Others (Larva, twig)	1 (2.3)	1 (3.3)
Indefinite	1 (2.3)	2 (6.7)

Complications resulting from bronchoscopic procedures were more common in children and were characterized by episodes of transient hypoxemia during removal.

## DISCUSSION

Individuals in the extreme age groups, specifically those below 3 years and above 70 years, are particularly susceptible to FB aspiration. We observed that food was the primary cause of aspiration in this series of patients across different age groups, which aligns with the findings reported in the medical literature. ^( 1 - 3 , 8 , 9 )^ Therefore, it is crucial to emphasize the importance of a careful and rational feeding process because multiple factors can contribute to aspiration events.

These factors include an individual’s level of consciousness, understanding of risk, presence of neuromuscular disorders, teething, chewing, and swallowing abilities, body positioning during feeding, type of food offered, exposure to potential risks (such as occupational hazards, inappropriate objects, or accidental situations), and habits of playing or moving while eating. It is important to note that aspiration events are not caused by a single isolated factor but rather by a combination of various factors. Managing these risk factors can significantly reduce the risk of aspiration.

In particular, young children are at a higher risk of FB aspiration because of their limited understanding of the situation and lack of supervision from guardians, who may be unaware of the risks or may fail to mitigate them ( *e.g* ., allowing children to talk, play, and run with food in their mouths). The teeth also play a significant role in food consumption. Typically, the molars of children are fully developed and capable of properly grinding food after 2 years of age. Before this age, their canines and incisors, which are sharp teeth, may not be effective in breaking down hard, spherical foods. Therefore, young children should avoid consuming such foods whenever possible. ^( 5 , 6 , 10 , 11 )^

Food plays a significant role in aspiration events in older individuals. Similar to young children, inadequate or absent dentition is often observed in older individuals. Neuromuscular factors, improper feeding techniques, inappropriate feeding positions, and fast eating contribute to the risk of aspiration. Occupational factors may also contribute to aspiration risk among adults.

Many of these aspiration incidents are preventable in both older individuals and children, highlighting the importance of raising awareness about the issue. In our sample, there was a higher proportion of older patients than children, which can be attributed to the aging population and increased life expectancy.

It is important to note that in cases of witnessed aspiration events, flexible bronchoscopy is a necessary and crucial diagnostic tool for excluding the presence of an FB, confirming the diagnosis of aspiration, and facilitating its removal. A history of FB aspiration is a significant indicator for bronchoscopy. In this study, approximately 90% of the patients with confirmed FB aspiration reported a history of choking and coughing. It is worth mentioning that studies have shown that less than 45% of patients with airway FBs exhibit symptoms such as cough, bronchospasm, or reduced breath sounds, and up to 25% of patients may have normal physical examination or imaging results. Radiographic findings such as atelectasis, hyperinflation, or radiopaque objects were present in less than 30% of cases. ^( 1 , 2 , 8 , 12 )^

In our study, we confirmed the presence of FBs in 70% of patients with clinical suspicion of aspiration, emphasizing the significance of a reported history of aspiration as a crucial factor in indicating the need for invasive investigation through bronchoscopy.

Indeed, patients with recent symptoms, such as cough, bronchospasm, or increased pulmonary secretions, along with a history of aspiration, should undergo further investigation, including imaging and bronchoscopy.

Several interesting aspects of endoscopic findings should be discussed. In children, the most common location of FBs was the larger bronchi, leading to significant airway obstruction. This can result in a higher frequency of hypoxemia and the need for emergency care compared to adults. Moreover, partial bronchial obstruction in children can lead to the development of a valve mechanism, increasing the risk of complications such as pneumothorax, pneumomediastinum, and further hypoxemia.

In adults, after an aspiration event, FBs may go unnoticed initially with minimal symptoms. This can result in recurrent or long-term infection of the affected lung ( Figure 1 ). Changes in inflammation and scarring can occur over time, leading to the formation of obstructive granulomas or scarring stenoses. These changes make manipulating and removing FBs via bronchoscopy more challenging. In some cases, multiple procedures were required for complete removal ( Figure 3 ).

These findings highlight the importance of early detection and intervention in children and adults to prevent complications and improve patient outcomes.

Choosing bronchoscopy equipment for FB removal is crucial for a safe and effective therapeutic approach. Flexible bronchoscopes and compatible accessories have proven to be highly effective and safe. It is important to select an appropriate bronchoscope compatible with the patient’s airway, ventilation, and anesthesia system during the procedure. Similarly, selecting suitable forceps for grasping and removing FBs is essential.

**Figure 4:**
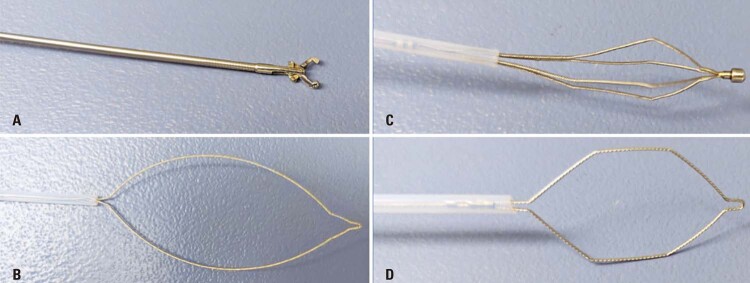
Flexible accessories for bronchoscope channel ≥2.0mm: A) Foreign body forceps “rat tooth” and B) Forceps; Flexible accessories for bronchoscope channel greater than or equal to 2.8mm: C) basket, D) polypectomy strap

There are various types of forceps available for use in flexible bronchoscopy, designed for different canal sizes, such as 2.0mm, 2.8mm, or 3.2mm ( Table 3 and Figure 4 ). Choosing an appropriate material, including the option of using a rigid bronchoscope, can enhance the safety and efficiency of the procedure. The availability of appropriate equipment in the procedural room is crucial.

**Table 3:** * Meat, vegetable fiber, fishbone, noodles, and bone fragments; # noodles. . Age, weight, cannula/laryngeal mask size and flexible bronchoscope

Age (years)	Weight (kg)	Cannula	Mask	Bronchoscope* (in mm)
1	5–10	4.0	1.5	3.1
1–5	10–20	4–5.5	2	3.1 or 4.2
5–10	20–30	5–6	2.5	3.1 or 4.2
10–15	30–60	5–7	3 or 4	4.2–5.5
Adult	>50	Over 6	4 or 5	4.2–6.2

While flexible bronchoscopy is often the preferred choice for FB removal, there is still a role for the rigid bronchoscope, particularly in cases where ventilation is compromised by the presence of devices within the airway or when forceps with better gripping ability are required. The experience and expertise of the bronchoscopist in working with both rigid and flexible bronchoscopes should be considered when selecting equipment for the procedure.

Performing bronchoscopy on a fasting patient is the preferred and safest approach whenever the patient’s clinical condition allows it. This is a standard practice in our service and is supported by medical literature. ^( 1 , 2 , 8 , 13 )^ Two anesthesia options during bronchoscopy are sedation with spontaneous ventilation and general anesthesia with intubation or a laryngeal mask. ^( 1 , 2 , 7 , 8 )^ In life-threatening emergencies where there is no adequate fasting time, bronchoscopy may need to be performed urgently. In such cases, measures to minimize the risk of aspiration, such as orotracheal intubation or protection using a rigid bronchoscope, should be implemented. ^( 8 , 13 )^

When performing flexible bronchoscopy through an orotracheal cannula, compatibility between the internal diameter of the orotracheal tube and the external diameter of the bronchoscopy equipment is necessary. This compatibility can make manipulation challenging. In our study, only one patient required a combination of rigid and flexible bronchoscopy techniques.

The interaction between the anesthesia and bronchoscopy teams is crucial for successful procedures. The bronchoscopist’s manipulation of the airway during the procedure can affect the patient’s ventilation, particularly in pediatric airways. Therefore, selecting a ventilation device that is appropriate for the caliber of the bronchoscope and the patient’s needs is important.

Table 3 provides a summary of device compatibility based on age, weight, and type of ventilation device, such as cannulas and masks. Laryngeal masks have been increasingly used for ventilation during anesthesia in FB removal procedures because they facilitate the process. However, endotracheal intubation tubes often impede the procedure, rendering manipulation and ventilation more challenging.

Utilizing a face mask for anesthesia may not consistently guarantee adequate ventilation, and there is a risk of displacement of the FB into the pharynx during its removal. Therefore, it is crucial to consider these factors and plan procedures accordingly to ensure patient safety.

Furthermore, it is important to note that all FBs in the airways below the nasal cavities should be removed orally. The nasal route should be avoided because of the risk of bleeding and the inability to pass through the nasal cavity.

Planning the materials for FB removal, including the availability of multiple flexible bronchoscope options, appropriate rigid bronchoscopes for different age groups, and various types of forceps, handles, and baskets, is essential. Nitinol-retractable baskets, commonly used for removing ureteral and renal stones, are compatible with bronchoscopes larger than 2.8mm. ^( 1 )^ Prior to manipulating the FB, a bronchial tree inspection was performed by instilling small aliquots of a diluted epinephrine solution (1:20,000 concentration, 1mL of adrenaline, and 19mL of isotonic sodium chloride solution). ^( 1 , 2 , 9 , 14 , 15 )^ This initial approach aimed to avoid fragmentation of the FB. In cases where the FB has been present for more than 4 weeks, removal may need to be performed in two stages, as previously described. During the first approach, a time limit of approximately 60 minutes was set, during which granulomas, secretions, and, if possible, the FB itself were removed. If the bronchial mucosa is highly inflamed and it is impossible to remove the FB, a second attempt can be made after 3–4 days, along with the use of systemic corticosteroids and antibiotics to control the inflammation and infection of the bronchial mucosa, which will facilitate subsequent manipulation. ^( 1 , 9 , 14 , 16 )^ In cases of long-term FBs associated with endobronchial scarring, removal can be performed after bronchial dilatation, as shown in figure 4 .

With the advancements in technology and therapeutic techniques, flexible bronchoscopy has become increasingly utilized to remove FBs in patients of different age groups. ^( 1 - 3 , 7 - 9 , 14 , 16 )^ However, this does not imply that rigid bronchoscopy should be disregarded; its use may be limited to specific cases where flexible bronchoscopy fails, appropriate equipment and accessories are lacking, or the bronchoscopist has extensive experience. ^( 16 )^

In our study, no fatal complications were encountered, and the most common adverse event was hypoxemia, which occurred in 5.1% of the procedures, predominantly in children. Other complications, such as hemorrhage or asphyxia during bronchoscopy, have not been reported in the literature. ^( 9 )^

We believe the high success rate of FB removal using flexible bronchoscopy can be attributed to several factors. First, prompt examination helps prevent distal impaction and the development of complications such as granulation, atelectasis, and hyperinflation. Second, ensuring a secure and effective coupling between the laryngeal mask or other ventilatory modalities and the bronchoscope allows for safe examination and manipulation of the airway. Additionally, the availability of diverse equipment, including a range of flexible video bronchoscopes with compatible accessories for different working channels, contributes to successful FB removal. The video modality enhances the synchrony between the operator using forceps and the bronchoscopist, and a well-trained team proficient in both rigid and flexible bronchoscopy techniques can alternate or combine modalities as needed, maximizing the chances of successful removal.

Considering these factors and employing appropriate techniques, the success rate of FB removal using flexible bronchoscopy can be significantly improved.

The training and availability of bronchoscopy resources in Brazil are limited, with only a few locations having the necessary infrastructure and resources to provide courses, simulators, and education in this field. Specialists are scarce, with approximately 120 bronchoscopists serving 5,570 municipalities in the country. Moreover, only approximately 6% of the cities (approximately 360 municipalities) have a population exceeding 100,000. However, even in these larger cities, infrastructure for complex procedures, such as FB removal in children, may still be lacking.

Given the intricacies and risks associated with managing FB aspiration, particularly in young children, it is crucial to emphasize preventive measures based on current scientific evidence. Healthcare professionals who frequently encounter such situations should disseminate this knowledge and educate the population about the risks and preventive measures associated with FB aspiration. ^( 1 , 7 - 10 )^

## CONCLUSION

A clinical history of foreign body aspiration serves as a valuable indicator for bronchoscopy and should be conducted promptly to prevent chronic inflammation and scarring complications of the bronchial mucosa. Organic foreign bodys particularly seeds, are commonly aspirated, and this occurrence is more prevalent in the extremes of age. Complications related to foreign body aspiration occur more frequently in children than in adults. Therefore, it is important to promote preventive measures against food intake within families. Flexible bronchoscopy, when performed by a skilled professional using suitable techniques and equipment, is effective and safe for managing patients with foreign body aspiration.
